# Genomic profiling of pan-drug resistant proteus mirabilis Isolates reveals antimicrobial resistance and virulence gene landscape

**DOI:** 10.1007/s10142-024-01419-7

**Published:** 2024-09-03

**Authors:** Sarah Soliman, Salah Abdalla, Amal Zedan, Shymaa Enany

**Affiliations:** 1 Trauma Intensive Care Unit, Zagazig University Hospitals, Zagazig, Egypt; 2 Department of Microbiology and Immunology, Faculty of Pharmacy, Suez Canal University, Ismailia, Egypt; 3 Department of Microbiology and Immunology, Faculty of Pharmacy, El Saleheya El Gadida University, El Saleheya, Egypt; 4 Department of Clinical Pathology, Faculty of Medicine, Zagazig University, Zagazig, Egypt; 5 Biomedical Research Department, Armed Force College of Medicine, Cairo, Egypt

**Keywords:** *Proteus mirabilis*, Whole genome sequencing, Antimicrobial resistance, Virulence

## Abstract

*Proteus mirabilis* is a gram-negative pathogen that caused significant opportunistic infections. In this study we aimed to identify antimicrobial resistance (AMR) genes and virulence determinants in two pan-drug resistant isolate “Bacteria_11” and “Bacteria_27” using whole genome sequencing. *Proteus mirabilis* “Bacteria_11” and “Bacteria_27” were isolated from two different hospitalized patients in Egypt. Antimicrobial susceptibility determined using Vitek 2 system, then whole genome sequencing (WGS) using MinION nanopore sequencing was done. Antimicrobial resistant genes and virulence determinants were identified using ResFinder, CADR AMR database, Abricate tool and VF analyzer were used respectively. Multiple sequence alignment was performed using MAFFT and FastTree, respectively. All genes were present within bacterial chromosome and no plasmid was detected. “Bacteria_11” and “Bacteria_27” had sizes of approximately 4,128,657 bp and 4,120,646 bp respectively, with GC content of 39.15% and 39.09%. “Bacteria_11” and “Bacteria_27” harbored 43 and 42 antimicrobial resistance genes respectively with different resistance mechanisms, and up to 55 and 59 virulence genes respectively. Different resistance mechanisms were identified: antibiotic inactivation, antibiotic efflux, antibiotic target replacement, and antibiotic target change. We identified several genes associated with aminoglycoside resistance, sulfonamide resistance. trimethoprim resistance tetracycline resistance proteins. Also, those responsible for chloramphenicol resistance. For beta-lactam resistance, only bla*VEB* and bla*CMY-2* genes were detected. Genome analysis revealed several virulence factors contribution in isolates pathogenicity and bacterial adaptation. As well as numerous typical secretion systems (TSSs) were present in the two isolates, including T6SS and T3SS. Whole genome sequencing of both isolates identify their genetic context of antimicrobial resistant genes and virulence determinants. This genomic analysis offers detailed representation of resistant mechanisms. Also, it clarifies *P. mirabilis* ability to acquire resistance and highlights the emergence of extensive drug resistant (XDR) and pan-drug resistant (PDR) strains. This may help in choosing the most appropriate antibiotic treatment and limiting broad spectrum antibiotic use.

## Background

*Proteus mirabilis*, a gram-negative rod-shaped bacterium, belongs to the Enterobacterales order within the Morganellaceae family. Considering all Proteus species, *Proteus mirabilis* (*P. mirabilis*) isolates were the most clinically significant and typically in charge of wound and most urinary tract infections acquired with patients in health care settings. According to recent studies, *Proteus mirabilis* has been found in 5–18% of instances of Gram-negative bacteremia (Chakkour et al. 2024). In Egypt, (57.8%) of Proteus isolates were found to be *P. mirabilis* (Ali et al. 2023). They are one of the most frequent causes concerning sepsis and nosocomial UTIs worldwide (Armbruster et al. 2018; Adamus-Bialek et al. 2013; Gomes Abreu et al. 2011).

*P. mirabilis* bacteria are opportunistic human pathogens. They are normally inhabitants of the human and animal gastrointestinal tract, skin, and oral mucosa. Their numbers can be increased easily in immunocompromised patients or individuals receiving antibiotic therapy (Armbruster et al. 2018; Kushwaha et al. 2014).

Moreover, *P. mirabilis* strains that are extensively drug-resistant (XDR) or non-susceptible to one drug in all but one or two antimicrobial classes (Magiorakos et al. 2012) and multidrug-resistant (MDR) or non-susceptible to one drug in three or more antimicrobial classes (Magiorakos et al. 2012), are extensively increasing, making it more difficult to treat infections caused by *P. mirabilis* (Li et al. 2022). This is mainly due to their known intrinsic resistance for nitrofurans, polymyxins (colistin), tigecycline and tetracycline (Girlich et al. 2020) and for their multiple acquired antimicrobial resistance to the β-lactams, carbapenems, fluroquinolones, aminoglycosides, trimethoprim/ sulfamethoxazole and wide range of antimicrobial classes (Girlich et al. 2020).

Due to the numerous virulence factors, such as adhesin, the creation of biofilms, the urease and hemolysin production, motility, swarming, and fimbriae-mediated adherence (Armbruster et al. 2018; Armbruster and Mobley 2012; Schaffer and Pearson 2015a) *Proteus mirabilis* commonly causes urinary tract infections, particularly in patients with catheters, which can lead to severe bloodstream infections with high mortality rates (Schaffer and Pearson 2015a*).*

Owing to this virulence characteristics and highly antimicrobial resistance profile of *P. mirabilis*, it is now a main concern to public health. Unfortunately. *P. mirabilis* research and whole-genome sequencing are now far less advanced than those for ESKAPE infections. As only 3,206 whole-genome assemblies were available in GenBank for *P. mirabilis* by 30 Nov 2023 (https://www.ncbi.nlm.nih.gov/datasets/genome/). While, for example, *Acinetobacter baumannii* (27,561 assemblies) and for *Klebsiella pneumoniae* (58,631 assemblies).

In the present study, we used MinION sequencing system (Oxford Nanopore Technologies, Oxford, UK) a second- generation sequencing aiming to describe the entire genome of *P. mirabilis* samples that are resistant to drugs that were taken from hospitalized patients. The investigation's goal was to determine how common antimicrobial resistance genes are, and virulence determinants of *P. mirabilis*. This may provide theoretical basis for clinical treatment and ideas for controlling nosocomial infection.

## Methods

### Isolation and growth conditions of samples

Two *P.s mirabilis* samples were used, one is isolated from wound sample and the second is from sputum sample. Samples were collected from hospitalized ICU patients. For study participation, we obtained an informed consent from patients. Blood agar and MacConkey agar were used to cultivate bacterial isolates as part of standard hospital laboratory protocols. *P. mirabilis* identification were based on growth characterization on blood agar and lactose non-fermenting growth on MacConkey agar in addition to microscopic examination. For more confirmed results, samples were also detected by means of the BioMerieux VITEK 2 technology. To extract DNA, the strains were sub-cultured on MacConkey agar at 37 °C for 24 h.

### Testing for antibiotic susceptibility

The minimum inhibitory concentrations (MICs) of fifteen antimicrobial drugs were automatically found using the VITEK 2 system, including amikacin, gentamicin, tobramycin, ciprofloxacin, trimethoprim/sulfamethoxazole, minocycline, imipenem, meropenem, ceftazidime, cefepime, aztreonam, piperacillin, ticarcillin, piperacillin/ tazobactam and ticarcillin/clavulanic. The Clinical and Laboratory Standards Institute (CLSI 2023) breakpoints for Enterobacterales were used to interpret the results. Nonsusceptibility to three or more of the following antibiotic classes is known as multidrug resistance: fluoroquinolones, aminoglycosides, trimethoprim/sulfamethoxazole, cephalosporins, and b-lactam inhibitor combinations (Magiorakos et al. 2012).

### Bacterial DNA extraction

For fast purification and high quality of microbial DNA, the genomics DNA is extracted using the PureLink™ Microbiome DNA Purification Kit following the manufacturer’s instructions. Verify that the extracted nucleic acids' quantity and quality adhere to the guidelines provided in the kit's user manual.

### Library preparation and oxford nanopore sequencing

The Rapid Barcoding Kit was used for the library preparation. By using the transposome in the Rapid Barcoding Kit. It adds barcoded tags on the cleaved ends of template molecules while cleaving them simultaneously. The DNA was prepared, fragmented, and then tagged. Pooling libraries combine equal volumes of normalized libraries. Libraries are cleaned up using SPRI beads to eliminate short library fragments and purify the DNA in the libraries, and then they are examined using a Qubit fluorometer. Sequencing run started according to Oxford nanopore manufacturing instructions on MinION Mk1C for instant data streaming, rapid result, and ultra-long reads making it possible to identify intricate structural variants that are challenging for short reads to detect, thereby promoting genomic assemblies.

The key benefit of Nanopore sequencing is the ability to generate ultra-long reads—over 2 Mb read lengths have been achieved. With the potential to reconstruct complicated genomes and provide a more comprehensive understanding of genetic variants, the ultra-long read lengths are more likely to span entire regions of structural variation and repetitive sequence.

### Assembly, annotation, and alignment of genome

Using FastQC, the Fastq read quality was evaluated. Reads that were too short or of poor quality were then eliminated by applying the NanoFilt tool. Additionally, adaptor sequences found in the reads were removed by applying Porechop_ABI. Flye was used for de novo assembly, polishing, and annotation of the filtered readings, Medaka and Prokka, respectively, utilizing epi2me bacterial genomics workflow (v. 0.3.0). Next, the quality of the resulting assembly was evaluated using Quast (Table 1). Next, circular maps of both isolates were predicted with Proksee viewer.

**Table 1:** Genome assembly statistics on PROKKA annotation server

Sample no	Bacteria_11	Bacteria_27
Chromosome size	4,128,657 bp	4,120,646 bp
Contig no	1	3
Accession no	SAMN42841177	SAMN42841178
GC (%)	39.15	39.09
N50	4,128,657	4,105,018
N90	4,128,657	4,105,018
L50	1	1
L90	1	1
No. OF CDS	4799	4913
No. of tRNA	84	84
No. of rRNA	22	22

### Antimicrobial resistant genes identification, virulence factors detection, phylogenetic tree and taxonomic classification

The identification of antimicrobial resistance genes (AMR) and virulence factors (VF) was carried out using Amrfinderplus, Resfinder and Abricate tools, utilizing the NCBI and VFDB databases. Subsequently, heatmap was generated using R (complexheatmap package). The taxonomic classification of the samples was performed using the Kraken database and Kraken2 tool. Also, for comparative analysis, multiple sequence alignment (MSA) was done using MAFFT. Following this step, for tree construction FastTree 2.1 tool was used, and we used iTOL for tree visualization.

### Compliance with ethical standards

The ethical board at Suez Canal University approved this investigation (No. 202202MH1). The experiment was carried out in compliance with all applicable regulations and guidelines.

## Results

### Antibiotic resistance profiles of P. *mirabilis* “Bacteria_11” and “Bacteria_27”

Antimicrobial susceptibility test results showed that both isolates were resistant to ticarcillin, ticarcillin/ clavulanic, piperacillin, piperacillin/tazobactam, ceftazidime, cefepime, imipenem, meropenem, aztreonam, amikacin, gentamicin, tobramycin, ciprofloxacin, minocycline, and trimethoprim/sulfamethoxazole.

### General characteristics of P. mirabilis “Bacteria_11” and “Bacteria_27” genomes

**Fig. 1:**
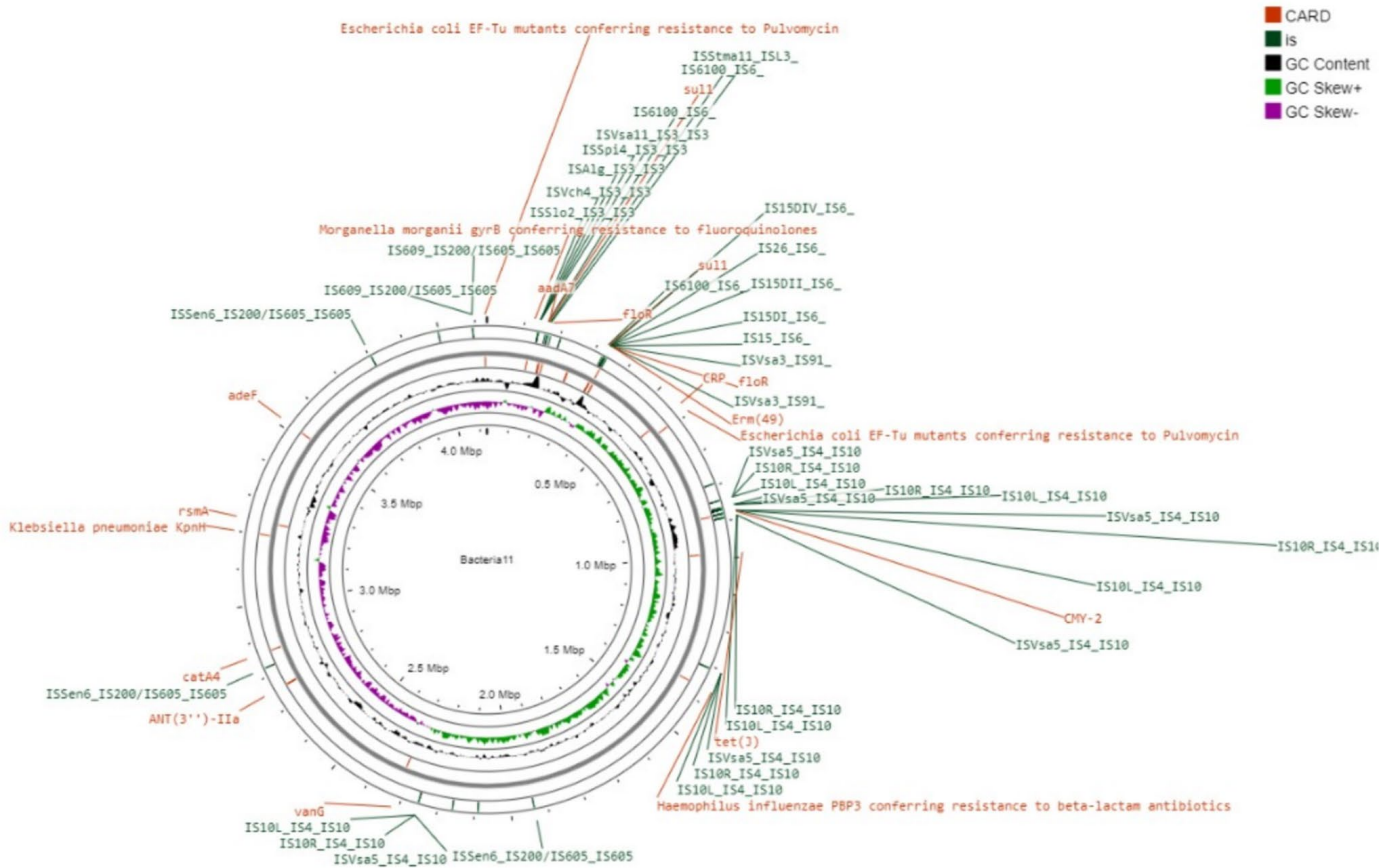
Circular Genome illustration of Bacteria _11 using Proksee viewer

**Fig. 2:**
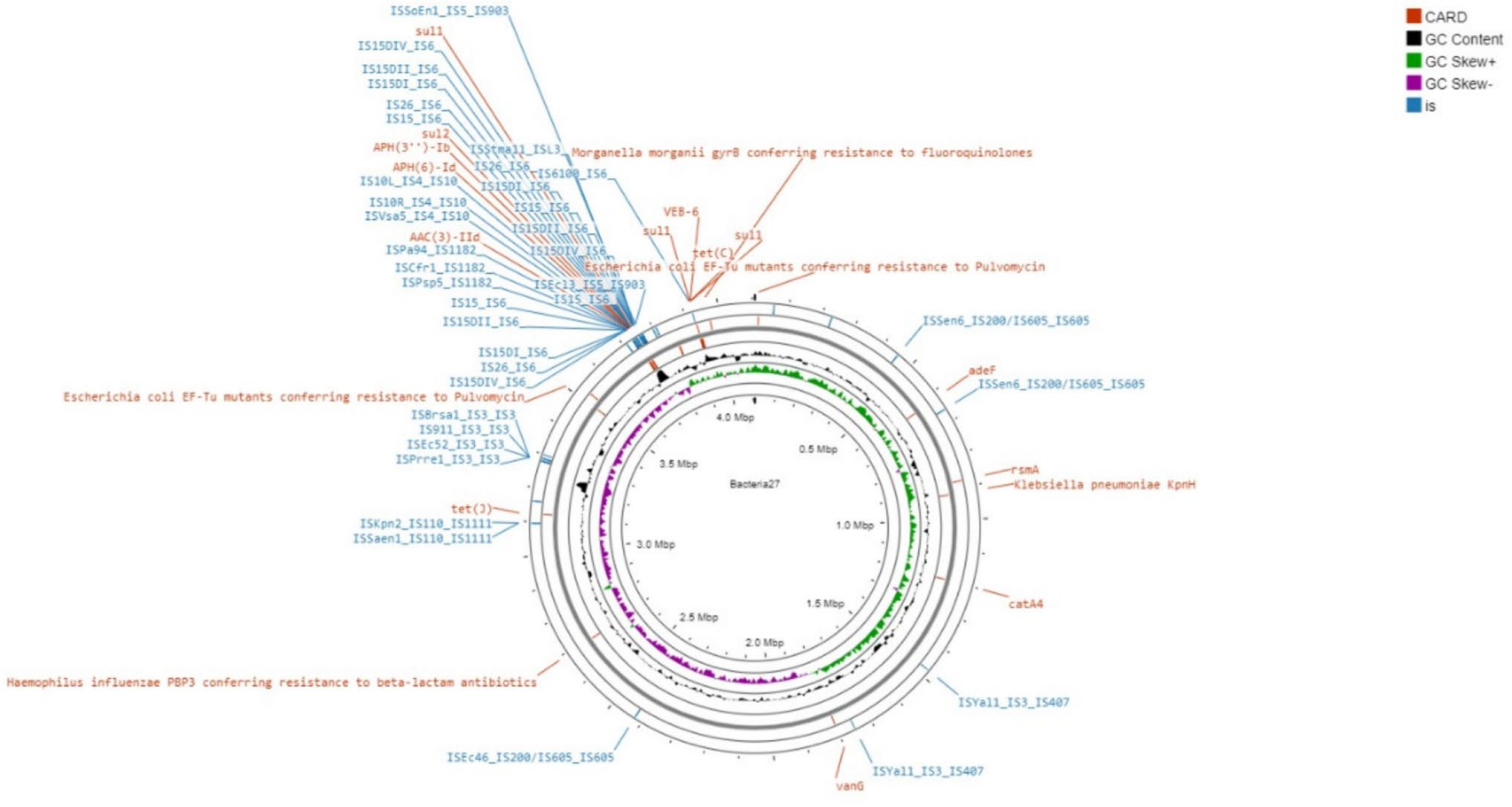
Circular Genome Illustration of Bacteria_27 using Proksee viewer

There was only the chromosome in *P. mirabilis* isolates, and no plasmid. Circular genome of the two isolates were shown in Figs. 1 and 2. All antimicrobial resistant genes were found on the chromosome, which had sizes of approximately 4,128,657 bp and 4,120,646 bp respectively, with GC content of 39.15% and 39.09%. “Bacteria_11” and “Bacteria_27” contained 4799 and 4913 coding genes respectively. Both isolates “Bacteria_11” and “Bacteria_27” sequence data are available in the NCBI GenBank databases under the accession numbers (SAMN42841177) and (SAMN42841178); respectively. We identified the presence of 43 and 42 antimicrobial resistance genes for both PM11 and PM27 respectively, accountable for the bacterial isolates' resistance to certain medication classes and up to 55 and 59 virulence genes were detected, respectively. Prokka additionally supplied the fundamental statistics for the genome assemblies' quality.

### Phylogenetic tree

**Fig. 3:**
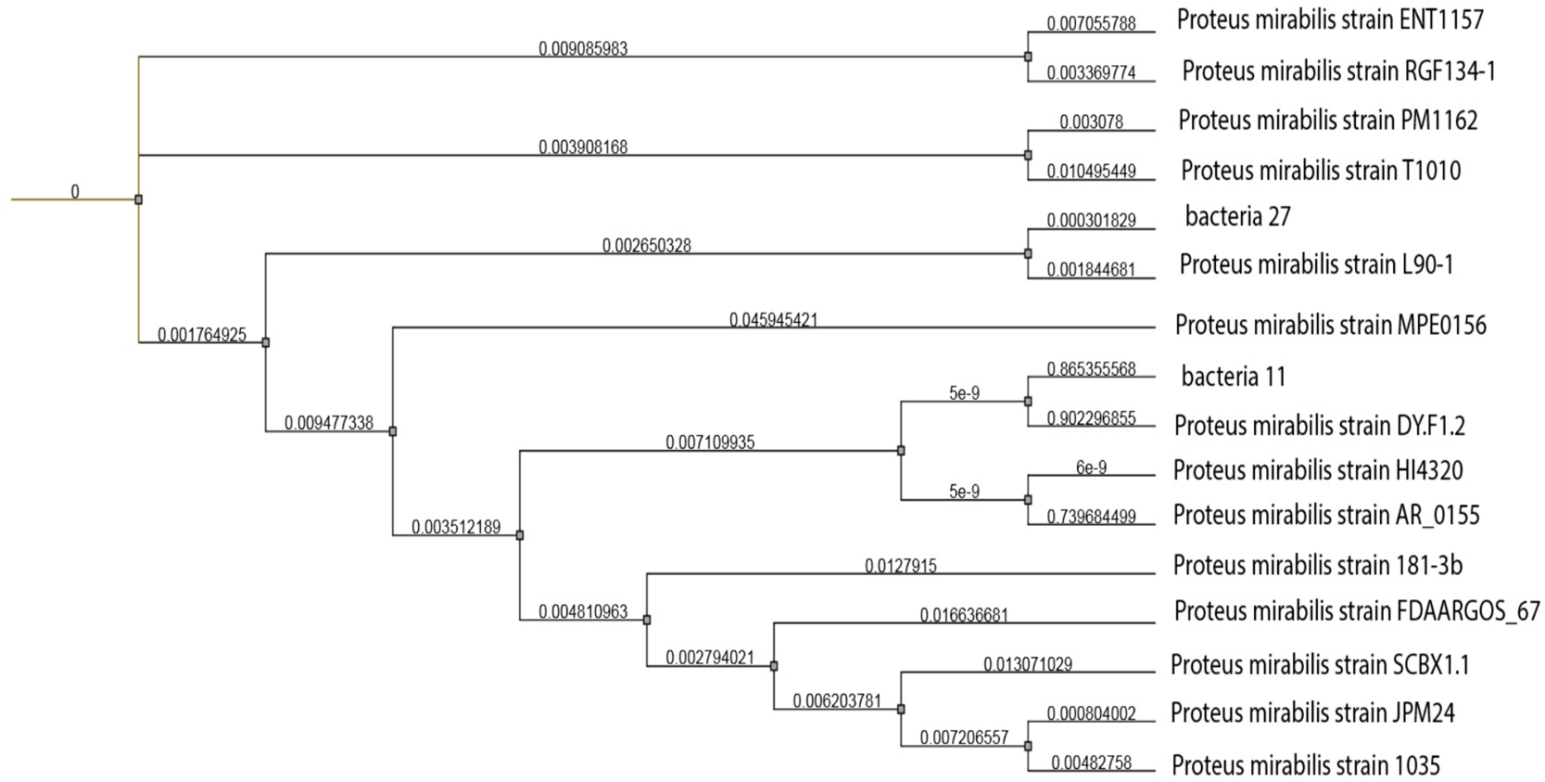
Phylogenetic Tree for “Bacteria_11” and “Bacteria_27”Proteus mirabilis strains. Multiple sequence alignment was conducted with MAFFT. Phylogenetic tree was constructed using FastTree and iTOL was used for visualisation. GenBank species names are given followed with isolate codes. Bootstrap values are shown on branches (1,000 replicate)

MAFFT was used for multiple sequence alignment. Phylogenetic tree was constructed by FastTree (Fig. 3). GenBank accession numbers of sequences were given then replaced by the species names and isolate codes. Bootstrap values are shown on branches (1,000 replicates). The closest strain to bacteria_27 was (Proteus mirabilis strain L90-1) taken from a stool specimen of human in Hangzhou, China. While for bacteria_11 was (*Proteus mirabilis* strain DY. F1.2) from a swine in Sichuan, China.

### Antimicrobial resistant genes identified in “bacteria_11” and “bacteria_27” isolates

To locate antimicrobial resistance genes in the assembled samples, we used the CARD AMR database (https://card.mcmaster.ca/), the NCBI Antimicrobial Resistance Genes database (combined with amrfinderplus) (v2023-04–17.1), and ResFinder 4.1 (https://cge.food.dtu.dk/services/ResFinder/).

**Fig. 4:**
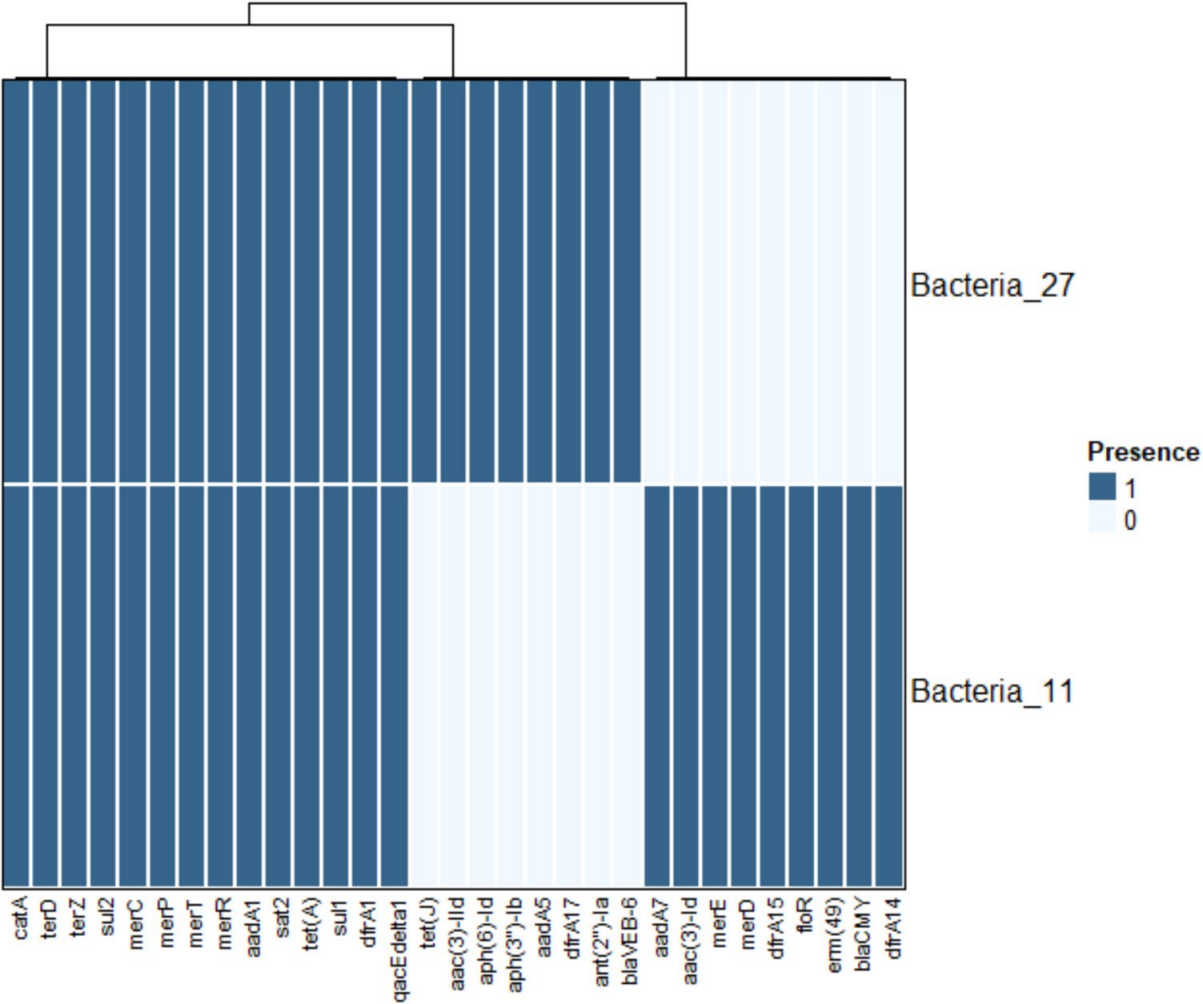
Heatmap of Antimicrobial Resistant Genes Identified in “Bacteria_11” and “Bacteria_27”

The data analysis revealed that the bacterial strains' resistance to various medication classes is caused by the presence of 43 and 42 antimicrobial resistance genes for “Bacteria_11” and “Bacteria_27”; respectively as illustrated in Table 2 and in the heat map (Fig. 4).

**Table 2:** AMR Genes Identified in Both Samples using amrfinder plus, CARD AMR and resfinder databases

Gene	Resistance Mechanism	Underlying Drug Resistance	Gene Function
*aadA1*	Antibiotic Inactivation	AMINOGLYCOSIDE	Nucleotidyltransferase
*aac(3)-Id*	Antibiotic Inactivation	AMINOGLYCOSIDE	N-acetyltransferase
*aadA7*	Antibiotic Inactivation	AMINOGLYCOSIDE	Nucleotidyltransferase
*aadA*	Antibiotic Inactivation	AMINOGLYCOSIDE	Nucleotidyltransferase
*ANT(2'')-Ia*	Antibiotic Inactivation	AMINOGLYCOSIDE	Nucleotidyltransferase
*AAC(6')-Ib10*	Antibiotic Inactivation	AMINOGLYCOSIDE	6'-N-acetyltransferase
*AAC(3)-IId*	Antibiotic Inactivation	AMINOGLYCOSIDE	N-acetyltransferase
*APH(6)-Id*	Antibiotic Inactivation	AMINOGLYCOSIDE	Phosphotransferase
*APH(3'')-Ib*	Antibiotic Inactivation	AMINOGLYCOSIDE	Phosphotransferase
*blaCMY*	Antibiotic Inactivation	Carbapenem; Cephalosporin; Cephamycin; Penam	Class C beta-lactamase
Haemophilus influenzae *PBP3* conferring resistance to beta-lactam antibiotics	Antibiotic Target Alteration	cephalosporin; cephamycin; penam	Penicillin-binding protein mutations
*blaVEB-6*	Antibiotic Inactivation	cephalosporin; monobactams	Class A beta-lactamase
*qacJ*	Antibiotic Efflux	DISINFECTANT AGENTS AND ANTISEPTICS	small multidrug resistance (SMR) antibiotic efflux pump
*tet(J)*	Antibiotic Efflux	Doxycycline, Tetracycline	tetracycline efflux MFS transporter
Escherichia coli *EF-Tu* mutants conferring resistance to Pulvomycin	Antibiotic Target Alteration	elfamycin	Elfamycin resistant
Morganella morganii *gyrB* conferring resistance to fluoroquinolones	Antibiotic Target Alteration	fluoroquinolone	fluoroquinolone resistant
*rsmA*	Antibiotic Efflux	fluoroquinolone; diaminopyrimidine; phenicol	resistance-nodulation-cell-division (RND)
*adeF*	Antibiotic Efflux	fluoroquinolone; tetracycline	resistance-nodulation-cell-division (RND)
*vanG*	Antibiotic Target Alteration	glycopeptide antibiotic	glycopeptide resistance gene cluster
*erm(49)*	Antibiotic Target Alteration	MACROLIDE	23S rRNA (adenine(2058)-N(6))-methyltransferase
*CRP*	Antibiotic Efflux	MACROLIDE	resistance-nodulation-cell-division (RND)
Klebsiella pneumoniae *KpnF*	Antibiotic Efflux	macrolide; aminoglycoside; cephalosporin; tetracycline; peptidE; rifamycin; disinfecting agents and antiseptics	small multidrug resistance (SMR)
Klebsiella pneumoniae *KpnH*	Antibiotic Efflux	macrolide; fluoroquinolone; aminoglycoside; carbapenem; cephalosporin; penam; peptide; penem	major facilitator superfamily (MFS)
*merR*	Metal	MERCURY	mercury resistance transcriptional regulator
*merT*	Metal	MERCURY	mercuric transport protein
*merP*	Metal	MERCURY	mercury resistance system periplasmic binding protein
*merC*	Metal	MERCURY	organomercurial transporter
*merD*	Metal	MERCURY	mercury resistance co-regulator
*merE*	Metal	MERCURY	broad-spectrum mercury transporter
*ArnT*	Antibiotic Target Alteration	PEPTIDES	pmr phosphoethanolamine transferase
*catA*	Antibiotic Inactivation	PHENICOL	O-acetyltransferase
*floR*	Antibiotic Efflux	PHENICOL	MFS transporter
*qacEdelta1*	Antibiotic Efflux	QUATERNARY AMMONIUM	SMR transporter
*sat2*	Antibiotic Inactivation	STREPTOTHRICIN	N-acetyltransferase
*sul2*	Antibiotic Target Alteration	SULFONAMIDE	Dihydropteroate synthase
*sul1*	Antibiotic Target Alteration	SULFONAMIDE	Dihydropteroate synthase
*terD*	Metal	TELLURIUM	Membrane protein
*terZ*	Metal	TELLURIUM	tellurium resistance-associated protein
*tet(A)*	Antibiotic Efflux	TETRACYCLINE	MFS transporter
*dfrA1*	Antibiotic Target Alteration	TRIMETHOPRIM	trimethoprim-resistant-dihydrofolate reductase
*dfrA14*	Antibiotic Target Alteration	TRIMETHOPRIM	trimethoprim-resistant-dihydrofolate reductase
*dfrA15*	Antibiotic Target Alteration	TRIMETHOPRIM	trimethoprim-resistant-dihydrofolate reductase
*dfrA17*	Antibiotic Target Alteration	TRIMETHOPRIM	trimethoprim-resistant-dihydrofolate reductase

Four resistance mechanisms were found in our analysis, which are caused by the identified resistant genes: antibiotic efflux, antibiotic inactivation, antibiotic target replacement, and antibiotic target change. Genes linked to aminoglycoside resistance were found, including *aadA1, aadA7, aadA5, aph(3)-Ib, and aph(6)-Id.* Also*, ant(2)-Ia, aac(6)-Ib,* and *aac(3)-Id* were identified as well. In Bacteia_11 genome, two copies of genes like *sul1* were detected and three copies were found in bacteria_27. Also, *sul2* was detected, which contributed to sulphonamide resistance. Additionally, we identified the genes *dfrA1, dfrA14, dfrA15,* and *dfrA17* linked to trimethoprim resistance. Tetracycline resistance proteins are designated as *tet(A), tet(J),* and *tet(C)*. In both genomes, two copies of the quaternary ammonium compound efflux SMR transporter*, qacl* and *qacEdelta1*, which confer resistance to disinfectants and antiseptics, were found. Additionally, another copy of *qacEdelta1* was found to be in bacteria_27 genome*.* We detected also *catA4* and *floR* responsible for chloramphenicol resistance. Bacteria_27 possessed *blaVEB* for beta-lactam resistance, but the other isolate, bacteria_11, possessed *blaCMY-2* gene. In both genomes, additional genes were identified as *sat2, erm* (49), *CRP, vanG, ArnT, terD*, and *TerZ*.

### Virulence characteristics of P. mirabilis isolates

Several virulence determinants contributing to the pathogenicity of *P*. *mirabilis* were found in both chromosomes. Virulence factors (VF) analysis of assembled samples was carried out using Abricate tool. The Virulence Factor Database was used to statistically assess the identity values ≥ 70% comparison results. Fifty-five and 59 virulence genes were detected in “Bacteria_11” and “Bacteria_27”; respectively.

Two genes that were found to be involved in the capsular polysaccharide were adhesion and elongation factor Tu (*EF-Tu*). Both isolates' genomes had the *luxS* gene, which is involved in quorum sensing in the biofilm forming mechanism.

The ability of *P. mirabilis* to swarm over solid surfaces is one of its primary characteristics. Genes involved in flagella formation were identified coding 5 different diverse of flagellum such as peritrichous flagella (*CheB*) and (*CheA, CheW, CheY, CheZ, MotA*), polar flagella (*nueA, FlmH*), flagella (*flgG, FlhC, FliP, FliI, FlgC, FlhA, FlgI*) flagella (*FliA, fliG, fliC, FlgH, Fli*M).

Another gene involved in the swarming process was (*RcsB*) a capsular synthesis two component system response regulator.

Numerous typical secretion systems (TNSSs) were present in the two isolates, including seven T6SS (*HcpA, Hcp, hcp-1, VipB, vipB/mglB, clpB/vasG,* and *hcp2/tssD2)* and two T3SS (*IpaH, bsaQ*).

The identified virulence genes located on the chromosome of both isolates were illustrated in Table 3 and Table 4.

**Table 3:** Virulence Genes Identified in Sample PM11

Isolate no	Virulence Factor Class	VF Accession number	Gene symbol	Identity %
Bacteria_11	ADHERENCE	WP_003028672	*tufA*	80.94
		WP_003028672	*tufA*	80.679
		WP_003028672	*tufA*	75
		WP_197535493	*htpB*	74.669
		NP_252312	*rpoS*	72.563
	Motility	WP_005160396	*fliM*	74.724
		WP_032902679	*CheA*	71.965
		WP_005160341	*fliA*	74.68
		WP_005160438	*flgG*	84.231
		WP_005164479	*CheY*	83.721
		WP_005164496	*FlhC*	83.333
		WP_005160341	*fliA*	83.122
		WP_005160381	*fliG*	81.707
		WP_011816618	*CheW*	81.046
		WP_011816591	*fliC*	80.46
		WP_042661456	*FliP*	80.176
		WP_026018001	*FliI*	79.646
		WP_005160434	*FlgH*	79.621
		WP_011816589	*fliC*	79.545
		WP_005160396	*FliM*	78.717
		WP_005168794	*FlgC*	77.612
		WP_011816614	*FlhA*	77.601
		WP_005300916	*flmH*	77.459
		WP_005164481	*CheB*	76
		WP_005164477	*CheZ*	73.585
		WP_042661458	*FlgI*	72.118
		WP_005164494	*motA*	71.284
		WP_016351249	*nueA*	70.281
	Immune-modulation	WP_014907233	*gndA*	85.928
		WP_005693586	*kdsA*	80.212
		WP_004175261	*ugd*	78.866
		WP_005632797	*rfaD*	78.065
		WP_005693459	*lpxC*	75
		WP_005693178	*galU*	70.035
		WP_005693459	*lpxC*	75.777
		WP_005694260	*gmhA/lpcA*	79.808
	Effector-delivery-system	NP_706650.1	*IpaH*	99.85
		NP_706650.1	*IpaH*	98.578
		NP_706650.1	*IpaH*	98.65
		NP_706650.1	*IpaH*	98.65
		NP_706650.1	*IpaH*	98.65
		NP_706650.1	*IpaH*	98.721
		WP_011705044	*HcpA*	89.535
		WP_011705706	*hcp*	88.953
		WP_001142947	*hcp-1*	78.488
		WP_043163044	*VipB*	76.423
		WP_000108140	*vipB/mglB*	75.891
		WP_000619136	*clpB/vasG*	70.482
		WP_001142968	*hcp2/tssD2*	83.14
	Exoenzyme	WP_002218234	*pla*	96.698
	Regulation	WP_002913007	*RcsB*	89.904
		NP_459678	*Fur*	86.395
		NP_461845	*RpoS*	79.939
	Biofilm	WP_001130227	*luxS*	76.687
	Antimicrobial-activity/Competitive-advantage	WP_002892069	*AcrAB*	75.38

**Table 4:** Virulence Genes Identified in Sample PM27

Isolate no	Virulence Factor Class	VF Accession Number	Gene Symbol	Identity %
Bacteria_27	ADHERENCE	WP_003028672	*tufA*	80.94
		WP_003028672	*tufA*	80.679
		WP_197535493	*htpB*	74.669
		NP_252312	*rpoS*	73.063
	Motility	WP_005160396	*FliM*	74.724
		WP_032902679	*CheA*	71.64
		WP_005160341	*fliA*	74.822
		WP_005160341	*fliA*	97.727
		WP_005164496	*FlhC*	95.122
		WP_005160341	*fliA*	90.196
		WP_005160438	*FlgG*	84.231
		WP_005160381	*fliG*	81.707
		WP_042661456	*FliP*	80.176
		WP_005160434	*FlgH*	79.621
		WP_011816589	*fliC*	79.545
		WP_005160396	*FliM*	78.717
		WP_011816614	*FlhA*	77.746
		WP_005168794	*FlgC*	77.612
		WP_005300916	*flmH*	77.459
		WP_011816589	*fliC*	77.273
		WP_005164481	*CheB*	76
		WP_005164477	*CheZ*	74.057
		WP_042661458	*FlgI*	72.118
		WP_005164496	*FlhC*	71.429
		WP_005164494	*MotA*	71.284
		WP_011816589	*fliC*	70.807
		WP_016351249	*NeuA*	70.281
		WP_005164474	*FlhB*	70.111
	Immune-modulation	WP_014907233	*gndA*	85.928
		WP_005693586	*kdsA*	80.212
		WP_005694260	*gmhA/lpcA*	78.125
		WP_004175261	*ugd*	78.093
		WP_005632797	*rfaD*	78.065
		WP_005693459	*lpxC*	75
		WP_005693178	*galU*	70.035
		WP_005693459	*lpxC*	76.295
		WP_005694260	*gmhA/lpcA*	79.808
	Effector-delivery-system	NP_706650.1	*IpaH*	98.65
		WP_011705044	*HcpA*	89.535
		WP_011705044	*hcp1*	89.535
		WP_001142947	*hcp-1*	89.474
		WP_011705706	*hcp*	88.953
		WP_011705706	*hcp*	87.791
		WP_001142968	*hcp2/tssD2*	83.14
		WP_001142968	*hcp2/tssD2*	81.977
		WP_001142947	*hcp-1*	78.488
		WP_001142947	*hcp-1*	78.488
		WP_001142947	*hcp-1*	77.907
		WP_043163044	*vipB*	76.22
		WP_000108140	*vipB/mglB*	75.891
		WP_011705044	*hcp1*	74.194
		WP_011705706	*hcp*	74.194
		WP_004188490	*bsaQ*	72.848
		WP_001142968	*hcp2/tssD2*	70
	Regulation	WP_002913007	*RcsB*	89.904
		NP_459678	*Fur*	86.395
		NP_461845	*RpoS*	80
	Biofilm	WP_001130227	*luxS*	76.687
	Antimicrobial-activity/Competitive-advantage	WP_002892069	*AcrAB*	75.475

## Discussion

*P. mirabilis* is recognized as an emerging healthcare concern due to its status as an opportunistic human pathogen, commonly found in the gastrointestinal tract, skin, and oral mucosa. Immunocompromised patients or those undergoing antibiotic therapy are particularly susceptible to its proliferation (Kushwaha et al. 2014).

Additionally, *P. mirabilis* exhibits intrinsic resistance to several antibiotics, including nitrofurans, tigecycline, tetracycline, and polymyxins (colistin) (Girlich et al. 2020; Stock 2003). Concern has escalated with the emergence of extended-spectrum β-lactamases (ESBLs), which confer resistance to cephalosporins (Stürenburg and Mack 2003), and the increasing prevalence of carbapenemase-encoding genes, limiting the effectiveness of existing antimicrobials (Adeolu et al. 2016; Firmo et al. 2020).

As human health depends greatly on the management of infectious diseases, particularly in light of the ongoing rise of MDR, XDR, or even PDR bacteria. Therefore, effective stewardship program implementation in any nation or region depends critically on an assessment of the local antimicrobial resistance trends and underlying resistance causes. That is why genomic profiling is highly beneficial in managing infections caused by pan-drug resistant *P. mirabilis.*

Herein, “Bacteria_11” and “Bacteria_27” were identified as pan drug resistant (PDR) *P. mirabilis* as they are non-susceptible to all tested agents in all antimicrobial categories (Magiorakos et al. 2012). They demonstrated substantial levels of resistance to various antibiotic classes in addition to their intrinsic resistance profiles. In a previous study, one of the first instance of PDR *P. mirabilis* in Egypt to be reported, 22.8% of isolates were MDR, 31.4% were XDR, and 8.5% were PDR (Algammal et al. 2021).

The phylogenetic analysis of the two isolates revealed that the closest strain to bacteria_27 was (*Proteus mirabilis* strain L90-1) (*genome* sequence ID: CP045257.1) with two plasmids. Sample was obtained from human stool specimen in China. Also, the closest strain to bacteria_11 was (*Proteus mirabilis* strain DY.F1.2**)** (*genome* sequence ID: CP046049.1) with a plasmid. It was taken from a swine in China as well. Many antimicrobial resistance genes were carried one the chromosome of both related strains while some were carried on the plasmids. This could be an indication that bacteria are borderless, and the global trade contribute to the spread of resistant bacteria worldwide. This strengthens the argument that antibiotic resistance is a global issue and adds to its complexity. Where a resistant bacterium emerges is irrelevant. In a globalized society, if it has a good chance of succeeding and becoming more and more popular, it might spread quickly to other regions of the world. The carriers of our two strains both were ICU patients. One was diabetic patient admitted to the hospital for above knee amputation as a result of infected diabetic foot. Unfortunately, patient died after 3 months of ICU- stay. The other was admitted as a post-operative patient, smoker, and hypertensive. He acquired chest infection and ventilated. He also passed away after 5 months of ICU- stay.

Our investigation revealed that the “Bacteria_11” and “Bacteria_27” resistance genes are all chromosomally situated. The four resistance mechanisms that caused this acquired resistance were antibiotic inactivation, antibiotic efflux, antibiotic target substitution, and antibiotic target modification.

We found that genes resistant to aminoglycosides were highly represented in both samples. The isolates exhibited resistance to gentamycin, amikacin, and tobramycin both phenotypically and genotypically. In both genomes, *aph (3'')-Ib, aph(6)-Id, aadA1, aadA7, aadA5, aac(3)-Id, ant(2")-Ia,* and *aac(6)-Ib*, were identified, which play a significant role in aminoglycoside resistance (Hua et al. 2020). These two isolates have high expression levels of genes like *aph (3′)Ib* and *aadA1*, which result in resistance to aminoglycoside drugs like streptomycin, kanamycin, and spectinomycin, which are rarely utilized in clinical settings and are not phenotypically assessed. In agreement with a previous study by (Yu et al. 2024) , these genes were highly represented in all tested *P.s mirabilis* isolates. In Egypt, this is not surprising, given that fosfomycin and aminoglycosides are often used to treat a variety of respiratory diseases, therefore the presence of this resistance pattern is expected (Mohamed et al. 2018). Moreover, this is in accordance with a recent study conducted in the same region (Zagazig) in Egypt except for isolates source (Tartor et al. 2021). They detected similar antimicrobial resistance (AMR) genes in three *P. mirabilis* strains isolated from both clinical and subclinical mastitis milk samples. These results underscore the principle of One Health and draw attention to the critical issue of the transfer of AMR genes between humans, animals, and the environment (Tartor et al. 2021).

*P. mirabilis* tested isolates were resistance to different beta-lactam antibiotic classes either penicillins (Ticracillin, Ticarcillin/ Clavulanic, Piperacillin, Piperacillin/Tazobactam), monobactams (Azetreonam) or broad spectrum cephalosporins (ceftazidime, cefepime). In fact, *P. mirabilis* producing (ESBL) genes is increasing aggressively in Egypt. A previous study reported that (57.6%) of the examined isolates produced (ESBLs) (ElTaweel et al. 2024). In our study the resistance may be assigned to the presence of extended spectrum beta-lactamase (ESBL) genes whether *blaCMY* gene in bacteria_11, a beta-lactamase that mainly occurs in *Klebsiella pneumoniae* and provides resistance to carbapenems, cephalosporins, penam, and cephamycins. Also, it is commonly found in *P. mirabilis*. As it was reported in another study that *blaCMY-2* was present in 22% of the isolates (Chalmers et al. 2023). Furthermore, *blaVEB-6* found in bacteria_27 which is a beta-lactamase commonly found in *P. mirabilis* (Zong et al. 2008) which has a role in resistance of cephalosporins and monobactams. In addition to the genes *kpnH, kpnF, rsmA* and *crp* that were found in both isolates, related to multidrug efflux pumps. Although Proteus by nature has reduced sensitivity to imipenem (Girlich et al. 2020), but these genes also may be the core reason for carbapenems (meropenem, imipenem) resistance as there were no detection for carbapenemases in both isolates. Although carbapenemase resistant *P. mirabilis* is definitely increasing worldwide (Girlich et al. 2020), the detected genes in previous studies were relatively low. In Egypt, a recent study found carbapenem resistant isolates were about (10.6%) (ElTaweel et al. 2024). Also, in Korean study, detection of carbapenemase genes were only in (15.6%) of isolated proteus samples (Yu et al. 2024). The World Health Organization (WHO) believes this to be a major public health problem since these antibiotics are used to treat diseases that are difficult to treat and because the number of bacterial strains that are resistant to them is rising and there are fewer therapeutic options available (Langford et al. 2023).

It was noticed the significant increase in infection caused by *P. mirabilis* in Egypt over the years especially after COVID era and the tremendous usage of antibiotic that contributed to this major range of resistant antibiotics(Negm et al. 2023).

We also figured out that there was no surprise for trimethoprim/ sulfamethoxazole resistance as this combination always used worldwide for urinary tract infection treatment, *Pneumocystis carinii* pneumonia, otitis media in infants and prostatic infections (Tuomisto et al. 2009; Kester et al. 2012). As *sul1* and *sul2* responsible for sulphonamide resistance were found repeated several times in both chromosomes and *dfrA1, dfrA14, dfrA15, dfrA17*, the reason for trimethoprim resistance also were detected. Also, a study conducted in Iran, reported the prevalence of *sul1* and *sul2* by (51.7%) and (56.7%); respectively (Human and Genom 2022). Similarly, this was found in the previously mentioned recent study (Tartor et al. 2021) which in turn emphasizes the concept of inter-species transfer between animals, the environment, and humans and again further underscores the One Health paradigm.

As β-lactams become resistant, usually quinolones offer a potential and comparatively safe alternative. In this study, quinolones (ciprofloxacin) resistance was detected phenotypically and genotypically by the presence of *gyrB* in both isolates, CRP, and multidrug efflux pump genes such as (*kpnH, kpnF, rsmA*). Unfortunately, *P. mirabilis* quinolones resistance is highly reported in Egypt. A significant (75.8%) quinolone resistance was found in the isolates that were examined (ElTaweel et al. 2024).

In addition to intrinsic resistance of *P. mirabilis* (Girlich et al. 2020), the presence of all those previously listed antimicrobial resistance genes (Table 2) in a single bacterial strain in addition to genes that provide resistance to antiseptics, diaminopyrimidines and chloramphenicol (*qacJ, KpnF, RsmA, catA,* and *floR*) is a huge concern and more worrying than expected. The isolates assessed in this study were obtained from nosocomial infections that were resistant to a wide variety of antibiotic groups in addition to having a high number of resistance genes. This implies that managing *P. mirabilis* infection with antibiotics poses significant challenges and entails a highly intricate process.

We may contribute the intrinsic resistant to tigecycline and some other antibiotics of *P. mirabilis* to the presence of the *AcrAB* transport system. The *AcrAB* efflux system transports hydrophobic substrates, such as detergents, dyes, and antibiotics, outside the cell directly with no accumulation in the periplasm (efflux) (Visalli et al. 2003).

Although our study is limited to two isolates only, the identified antimicrobial resistant genes may be present in many other Proteus isolates from the same region. This might help in choosing alternative therapeutic plans for treating hospital acquired infections from the beginning. As starting with initial loading dose followed with treatment period for 7 to 14 days. Also, combination therapy may be considered in clinical practice.

Alternatively, many virulence factors, such as the genes responsible for adhesion and swarming ability, are encoded by bacteria_11 and bacteria_27.

It is known that *P. mirabilis*, like other bacteria, uses flagella to swim through liquids and towards chemical ingredients. No surprise that both isolates have the main virulence genes encoding the flagellar components including the two flagellins *flic1* and *flic2* which comprise the wipe structure of flagellum (Schaffer and Pearson 2015a). As motility and swarming ability on solid surfaces is a main feature in *P. mirabilis* a lot of genes involved in flagella formation were identified coding 5 different diverse of flagellum (Armbruster and Mobley 2012).

Pathogenicity of *P. mirabilis* is highly detected in our isolates. Genes contribute to adherence were highly detected in both samples. It is known that the bacterial adherence of *P. mirabilis* is a crucial stage in the colonization and development of infections (Hasan 2020) Also, *fliA, fliM, cheA* and a lot of other genes contributing to bacterial swarming and motility were highly detected in both samples. According to Kuan et al. the primary virulence factor of *P. mirabilis* that affects the invasion and dissemination of infection in urinary tract segments is its motility (Kuan et al. 2014). This bacterium swarming motility increases the expression of its pathogenicity (Kotian et al. 2020) Furthermore, one of the key factors for *P. mirabilis* pathogenicity is biofilm formation*. P. mirabilis* expresses several virulence factors necessary for the formation of biofilms. Such as adhesion proteins, quorum sensing molecules, lipopolysaccharides, efflux pumps, and urease enzymes are a few examples of these variables (Schaffer and Pearson 2015b) Unfortunately, Crystalline biofilm-embedded bacteria develop a strong resistance to both the immune system and traditional antimicrobial drugs.

Very few studies were concerned with *P. mirabilis* acquired infection, especially in Egypt. Our study highlights *P. mirabilis* ability to acquire resistance for different antimicrobial classes used commonly in clinical aspects. In addition to its large virulence profile, to defeat host defensive mechanisms. In this study we were allowed to know the behaviour, adaptation, and pathogenicity of these bacteria. Also, we were able to pinpoint acquired resistance genes to make better informed antibiotic treatment choices.

Antibiotic resistance genes, virulence factors, and the all-other isolates traits that may be inferred from genomic sequences provide helpful information on the range of *P. mirabilis* pathogenicity and antimicrobial resistance acquired by them. Unfortunately, this study is limited to two isolates only, but it demonstrates the huge range of acquired antimicrobial resistance of *P. mirabilis* species. Further research is needed in the future to potentially uncover more of these transfer events, particularly in hospital-acquired illnesses. Our analysis of the pathogenicity and antibiotic resistance of these isolates could contribute to the future studies of multidrug-resistant *P. mirabilis* emergence and, hopefully, could lead to effective treatment strategies.

The availability of more whole genomes sequenced especially those sequenced with long-read technologies such as Nanopore will make it easier to comprehend how *P. mirabilis* acquires antimicrobial resistance.

## Conclusions

*P. mirabilis* isolates carried multiple antimicrobial resistant genes with different resistance mechanisms and a wide range of virulence determinants. This study highlights antimicrobial resistance genes that contributes to narrowing therapeutic options against *P. mirabilis.* This may help in choosing the most appropriate antibiotic treatment and limiting broad spectrum antibiotic use. Also, this contributes to reduction of using unnecessary antibiotics which slower the expansion of antimicrobial resistance.

## Data Availability

No datasets were generated or analysed during the current study.
